# Creek Beds and Cape Hares: Spatial and Seasonal Determinants of Caracal Movement and Diet in a Hyperarid Desert Ecosystem

**DOI:** 10.1002/ece3.73117

**Published:** 2026-02-17

**Authors:** Adi Barocas, Yaron Weisbein, Eli Geffen

**Affiliations:** ^1^ Hula Research Center Tel‐Hai University of Kiryat Shmona & the Galilee Kiryat Shmona Israel; ^2^ MIGAL–Galilee Research Institute Kiryat Shmona Israel; ^3^ Ze'ev Brande 5 Petah Tikva Israel; ^4^ School of Zoology Tel Aviv University Tel Aviv Israel

**Keywords:** arid ecosystems, caracal, carnivore conservation, habitat selection, human‐wildlife interface, resource selection function

## Abstract

Native mesopredators face challenges adapting to landscapes modified by human activities, particularly in arid regions, where resources are naturally limited and ecological dynamics are sensitive to disturbance. The caracal ( *Caracal caracal* ), a behaviorally flexible predator found across Africa and Asia, offers insights into how such carnivores navigate the balance between natural habitat requirements and anthropogenic pressures. We investigated habitat selection and dietary patterns of caracals in southern Israel's hyperarid Arava Valley using radiotelemetry data from 10 collared individuals (*n* = 75 tracking days) and scat samples collected during 1986–1987, complemented by ranger observations spanning the four subsequent decades. Resource Selection Function analyzes revealed pronounced sex‐specific and seasonal patterns of habitat selection within observed areas of use. Males exhibited larger areas of use than females, whereas habitat selection differed primarily through sex‐dependent responses to topography, elevation, hydrological features, and anthropogenic drivers rather than consistent differences in overall terrain preferences. These sex‐specific differences were evident in both seasons but were most pronounced during the dry season, suggesting increased habitat segregation under conditions of heightened resource limitation. Both sexes strongly selected for proximity to creek beds across seasons, likely reflecting high natural prey availability—particularly Cape hares, as well as more buffered microclimatic conditions. Caracals demonstrated context dependence in their response to human infrastructure—avoiding paved roads during the dry season, showing seasonal shifts in their use of dirt roads, and selecting areas closer to agricultural fields primarily during the dry season, especially females. Dietary analysis indicated a strong reliance on wild prey, dominated by Cape hares, small rodents, and desert partridge. Only 11.3% of fecal samples contained anthropogenic food items. Long‐term observational data confirmed that both caracals and their primary prey exhibited significant spatial association with creek beds. Together, these complementary lines of evidence suggest that natural prey availability remains the dominant driver of caracal habitat selection in this resource‐limited system. Conservation efforts should prioritize the protection and connectivity of creek bed habitats to support the persistence of caracals and other native species in increasingly human‐dominated arid landscapes.

## Graphical abstract

This study examined habitat selection and diet of caracals in Israel's hyperarid Arava Valley using radio‐telemetry data, scat analysis, and four decades of observations, revealing that caracals strongly prefer creek bed habitats year‐round while showing sex‐specific differences in home range size and seasonal responses to human infrastructure. The dietary analysis demonstrated that caracals primarily consume wild prey (Cape hares, small rodents, and desert partridge) rather than human‐provided food sources, with both predators and prey showing strong spatial association with creek beds. The findings suggest that natural prey availability drives caracal habitat selection in this resource‐limited environment, highlighting the importance of protecting creek bed habitats and maintaining connectivity for conservation of desert carnivores.

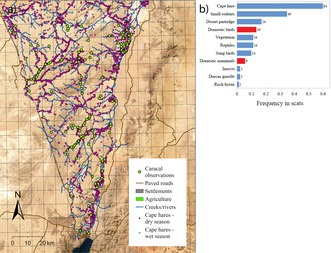

## 1 Introduction

Mammalian carnivores are increasingly forced to live near human habitation and infrastructure (Chapron et al. 2014). The immediate responses of these species to such disturbances are typically behavioral (e.g., altered habitat selection, activity, or vigilance; Tuomainen and Candolin 2011), and thus behavioral plasticity is essential for population persistence in changing environments (Wong and Candolin 2015). A key determinant of landscape use by carnivores is the perception of risk, whereby individuals must balance the rewards (e.g., attaining food or shelter) with potential costs (e.g., predation; Frid and Dill 2002; Johnson et al. 2020). In some disturbed landscapes in which humans are ecologically perceived as apex predators (Clinchy et al. 2016), the consequent changes in animal behavior have important implications for wildlife communities and ecosystem processes (Suraci et al. 2019).

Arid environments have expanded globally in recent decades (Lewin et al. 2024). Both human activities and the expansion of commensal species that accompany them have driven ecological changes in desert landscapes, including declines in native species. Despite the increase in human populations that depend on arid ecosystems, for logistic and cultural reasons these areas remain relatively understudied (Pyšek et al. 2008; Brito et al. 2009). Specifically, our knowledge of the distribution and spatial behavior of mammalian carnivores in desert areas is deficient.

Human activities in arid and semi‐arid zones drive significant changes in the habitat selection and behavior of medium‐sized carnivores (e.g., jackals, foxes, cats, and civets), including temporal niche shifts, spatial adjustments to human‐dominated landscapes, and increased interspecific competition, with generalist species showing higher adaptability compared to specialists (Vanak and Gompper 2010; Reshamwala et al. 2018; Devarajan and Vanak 2020; Ganguly et al. 2024).

The caracal ( *Caracal caracal* ) is a medium‐sized ecological generalist, sometimes considered a conflict species due to its interactions with humans in anthropogenic landscapes. In urban‐dominated regions, caracals preferentially forage near the urban‐natural interface, employing spatial risk mitigation strategies to navigate human activity. In contrast, caracals in wildland regions distinctly avoid human infrastructure. Although caracals display some limited temporal shifts, such as increased nocturnality near human settlements, they rely more on spatial strategies to adapt to human presence (Leighton et al. 2021; Serieys et al. 2023).

As highly adaptable generalists, caracals exploit a wide array of prey resources, but typically focus on the most abundant native species (Avenant and Nel 1998; Pohl 2015). In a survey of diets throughout the species' range, caracals were found to consume 151 different prey species across multiple taxonomic groups, including mammals, birds, amphibians, reptiles, arachnids, insects, and, uniquely among the studied felids, millipedes. The most frequent prey items consumed were lagomorphs, artiodactyls, hyraxes, livestock, and smaller carnivores (Parchizadeh et al. 2023).

Previous studies have shown that caracals are highly adaptable and select their microhabitats based on prey availability and environmental conditions. They commonly adapt their prey selection to the availability of diverse prey species, most frequently mammals, in the landscape (Bothma and Le Riche 1994; Avenant and Nel 2002). In some regions, caracals prefer coastal zones and areas with vegetative cover, such as fynbos and agricultural boundaries (Ramesh et al. 2017; Serieys et al. 2023). Physical barriers such as fences and other types of urban development have been shown to fragment caracal populations, limiting connectivity in certain regions (Leighton et al. 2023). Interestingly, caracals thrive in farming areas, where they may experience ecological release due to lower presence of large carnivores (Drouilly and O'Riain 2019).

Recent studies have indicated that caracals persist in diverse environments, including large protected areas near human habitation (Serieys et al. 2019; Leighton et al. 2020), small reserves surrounded by urban development (Schnetler et al. 2021), and heavily modified residential areas (Nattrass and O'Riain 2020). However, these urban areas may act as ecological traps, exposing caracals to risks such as toxic substances (Serieys et al. 2019). Taken together, these studies indicate that caracals exhibit remarkable adaptability in human‐dominated landscapes, with their proximity to humans presenting both opportunities and significant challenges.

Caracal populations in Asia and the Middle East face significant regional threats, leading to a regional Near Threatened classification due to habitat loss, fragmentation, and human–wildlife conflict. Studies in Iran suggest that caracals occupy a wide variety of dry habitats, including semi‐arid mountainous woodlands and plains, but mortality records show that caracals are at risk from vehicle collisions, poaching, and retaliatory killing by traditional pastoralists following occasional predation on domestic small stock (Moqanaki et al. 2016). Furthermore, research in Southwestern Turkey indicates that its isolated population favors complex landscapes like pine woodlands with high habitat heterogeneity, and actively avoids highly forested areas and human disturbance (Ilemin and Gürkan 2010; Ilemin et al. 2023). These findings are further supported by species distribution models from Israel, indicating that caracals favor grasslands, semi‐arid zones, and agricultural mosaics, and generally avoid urban environments (Hadad et al. 2025). These regional findings provide further evidence for the caracal's adaptability and preference for open landscapes while navigating increasing anthropogenic pressures across its range.

Here, we investigated the spatial ecology of a caracal population inhabiting the periphery of small agricultural communities in the hyper‐arid region of southern Israel. Employing telemetry data and a Resource Selection Function (RSF; Boyce et al. 2002) framework, we tested two competing hypotheses. The first posited that caracals preferentially selected areas near human settlements, infrastructure, and agricultural lands, potentially drawn by anthropogenic food availability. The alternative hypothesis posited that their spatial behavior was more strongly influenced by natural landscape features—such as creek beds and gently sloping terrain—where encounters with natural prey are more likely. To further elucidate the foraging patterns inferred from spatial data, we conducted fecal diet analyzes. To contextualize these findings within a broader spatio‐temporal framework, we also incorporated observational data spanning four subsequent decades, collected by rangers of the Israel Nature and Parks Authority (INPA). This dataset documents sightings of both caracals and their primary prey, the Cape hare ( *Lepus capensis* ), across southern Israel.

## 2 Methods

### 2.1 Research Area

**FIGURE 1:**
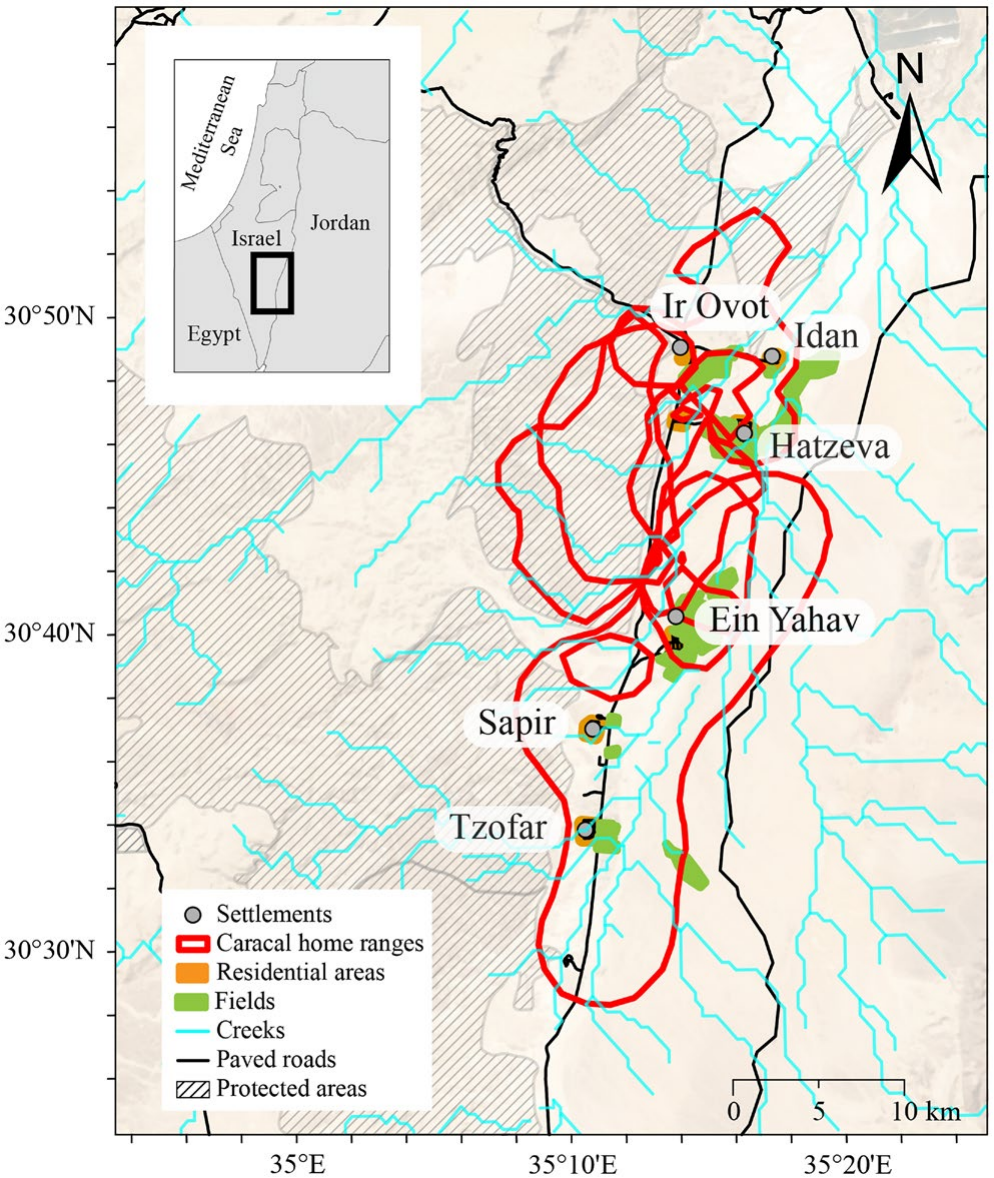
Map of the research area, including areas of space use by caracals captured and fit with VHF collars between 1986 and 1987 in southern Israel.

We conducted the study between 1986 and 1987 in the northern Arava Valley, a region stretching approximately 80 km in length in southern Israel (Figure 1). This is a climatically and geographically hyperarid region receiving an annual rainfall of about 40 mm, with an average minimum temperature of 7°C in winter, an average maximum temperature of 42°C in summer, and high evapotranspiration rates. The total human population of agricultural settlements in the region numbers nearly 6000, in addition to an unknown number of foreign national workers. Agriculture is the prominent economic activity, comprising date orchards, seasonal vegetables, flowers, dairy production, livestock rearing, and aquaculture (Lewin et al. 2021).

Outside agricultural and residential areas, the Arava valley is comprised of different habitats, namely alluvial fans, sand dunes, semi‐stable sands, salt marshes, and wadi beds. The wider wadi beds feature reduced vegetative cover. Lower areas above groundwater feature desert‐adapted bushes and trees such as acacias ( *Vachellia tortilis* ), Arabian boxthorn (*Lycium shawii*), jujube (*Ziziphus spina‐christi*), and tamarix (*Tamarix nilotica*) in addition to occasional annuals that grow following the rare flooding events. Depending on precipitation and flooding, wadi beds with bushy cover can create microclimates suitable for locally adapted rodents and reptiles (Shanas et al. 2006).

The local carnivore community includes the Arabian wolf ( *Canis lupus arabs* ), the striped hyena ( *Hyaena hyaena* ), the caracal, Ruppell's fox (*Vulpes rueppelli*), and the wild cat ( *Felis silvestris* ). Human‐associated species are the red fox ( *Vulpes vulpes* ) and golden jackal ( *Canis aureus* ; Mendelssohn and Yom‐Tov 1999; Cohen et al. 2013; Magory Cohen et al. 2013; Barocas et al. 2022; Lewin et al. 2023).

### 2.2 Animal Capture

We captured caracals using 40 × 40 × 100 cm cage traps (Tomahawk Live Trap Company, Tomahawk, WI, USA), baited with dead chickens, in a 1500 km^2^ area in the northern Arava valley (Figure 1). Each trap session comprised 10 traps, deployed along a transect. Traps were left open during the night and examined each morning. They were deployed in shady spots and beneath trees and secured with rocks and heavy branches. Captured individuals were sexed, weighed, measured, ear‐tagged, and fitted with collars with VHF telemetry tags (110–150 g; 1%–2% of caracal's body weight). All animal handling procedures complied with permits from the Israel Nature and Parks Authority.

Animals fitted with a radio‐collar were subsequently followed for triangulation fixes. We used a Merlin 12 VHF receiver (Wildlife Materials Inc.), an Omni directional antenna (Telonics Inc.) for detection with a vehicle and a three‐element folding Yagi antenna (Wildlife Materials Inc.) for detections on foot. Each focal animal was followed continuously about once a month by 4 × 4 car and by foot for 24‐h long sessions, conducted approximately once a month over a period of one to 2 years (Table S1). This approach provided monthly snapshots of individual movement rather than continuous long‐term tracking. Individual caracal locations were registered on a topographic map every 30 min. The majority of locations were confirmed visually using either binoculars, a telescope, or a night vision apparatus. When visual confirmation was not possible, triangulation of the VHF signal was used.

### 2.3 Habitat Covariates and Space Use Estimation

To calculate caracal observed areas of use, we utilized kernel utilization density, a probabilistic method that estimates an animal's home range by creating a bivariate probability density function, in which areas with higher densities of observations receive higher probability values, thus producing a utilization distribution that represents the relative frequency of space use (Seaman and Powell 1996). We delineated 50%, 90%, and 95% contours. We present home range areas for the 90% and 95% contours to account for the variability of habitat available to each individual and to mitigate the effects of single long‐distance movements and outlying points (Kie et al. 2010; Figure S1). We used the ‘*kud*’ function of the ‘*adehabitatHR*’ package (Calenge 2006) in the R computing environment (R Core Team 2020). To facilitate convergence we specified the type of kernel as bivariate normal (Worton 1989), the extent parameter to 1 and the smoothing parameter (h) to 1000.

**FIGURE 2:**
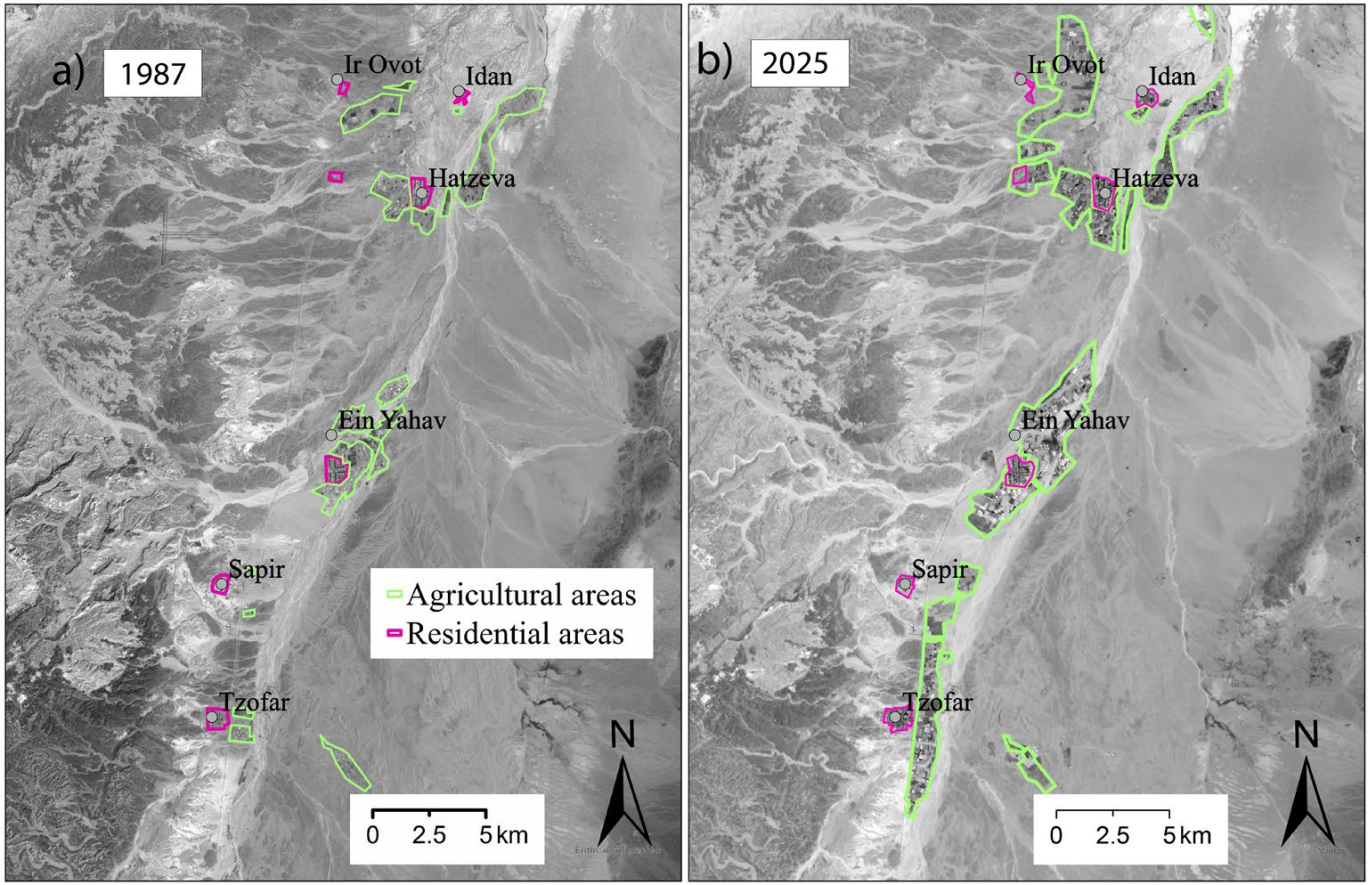
Comparison of the research area's landscape topography, creeks, and human activity between 1987 (a) and 2025 (b).

To assess the influence of topographical covariates and human activity on caracal space use and movement, we developed GIS layers quantifying distance of individuals locations from anthropogenic features, and distance from paved and dirt roads. We did not separately quantify distance from residential areas, agriculture and military bases because these distances are highly correlated in the research area (Barocas et al. 2018). Rather, we included in the models the distance from agricultural areas, which were the most prevalent human‐impacted landscape in the region. We digitized agricultural areas and residential areas from a satellite image dated April 1987 to ensure that the anthropogenic features in our model matched the landscape available to the caracals during the tracking period (Figure 2). We also obtained spatial data on paved and dirt road from the INPA, and linear features of rivers and creek beds from the free flowing rivers dataset (Grill et al. 2019). To quantify elevational and topographical variation within caracal observed use areas, we developed layers describing elevation, slope, aspect and topographic position (Table 1). All layers had pixel resolution of 30 m. To ensure temporal consistency between historical and contemporary landscape features, we compared creek patterns and topography from the 1987 satellite image with a contemporary Landsat 8 image from July 2025 (Earth Resources Observation and Science (EROS) Center 2020). Despite an increase in agricultural areas, the topography, road structure, and creek outlines remained nearly identical (Figure 2). We therefore used contemporary data for all layers except agricultural areas, which we derived from the 1987 image to maintain temporal alignment with our study period.

**TABLE 1:** Description and sources of spatial variables used as predictors of caracal space use using resource selection functions (RSF).

Name	Description	Min, max	Source
Settlement distance	Euclidean distance (km) from settlements in 1987	0, 21.9	INPA, satellite images of Jordan, Egypt
Field distance	Euclidean distance (km) from agricultural areas in 1987	0, 11	
Main road distance	Euclidean distance (km) from paved roads	0, 7.8	INPA, satellite images of Jordan, Egypt
Dirt road distance	Euclidean distance (km) from dirt roads	0, 3.8	INPA, satellite images of Jordan, Egypt
Creek distance	Euclidean distance (km) from dry creeks	0, 13.9	FFRivers dataset (Grill et al. 2019) with additional modeling using ArcHydro tools (Maidment 2002) on 1987 satellite image
Elevation	Value of a Digital Elevation Model (DEM) for each cell	0, 173	Aster GDEM, https://asterweb.jpl.nasa.gov/gdem.asp
Slope	Rate of elevation change among cells in degrees	0, 25.6	Developed from DEM
Aspect	The downslope direction of the maximum rate of change in value from each cell to its neighbors	0, 358	Developed from DEM
TPI	Topographic position index—calculated as the difference between the elevation of a cell and the mean elevation of its neighboring cells. Negative values represent valley bottoms	−15.4, 17.21	Developed from DEM

### 2.4 Resource Selection Functions

To investigate the factors that influence space use by caracals at the scale of space use areas, we employed a resource selection function (RSF) framework (Boyce et al. 2002). Based on seasonal variation in precipitation, we partitioned location data into dry (May 1—October 30; mean precipitation = 9 mm) and wet (November 1—April 30; mean precipitation = 30 mm) seasons. Areas within each individual's seasonal use areas were considered available habitat.

Following a third order selection design (Johnson 1980), we generated random (available) points within each individual's observed area of use, totaling double the number of observed (used) locations, and extracted habitat covariates for each used and available point (Figure S1). For the RSF analysis, to account for pseudo‐replication arising from repeated locations of the same individual, as well as spatiotemporal autocorrelation within tracking periods, we fitted binomial mixed‐effects logistic regression models using a generalized linear mixed model (GLMM) with a logit link (Harrison et al. 2018; Muff et al. 2020). To account for pseudo‐replication arising from repeated locations of the same individual, as well as spatiotemporal autocorrelation within tracking periods, we included caracal identity and tracking date as random effects.

RSF therefore reflect third‐order habitat selection within discrete, short‐term windows of activity rather than continuous movement processes. We specified random intercepts to capture variation in habitat availability among individual use areas, but excluded random slopes to ensure model convergence and avoid overparameterization. Finally, to address collinearity among predictors, we calculated Variance Inflation Factors (VIF) for each predictor (Zuur et al. 2010) and retained only predictors with VIF ≤ 10.

Because there is evidence that home range size may vary between males and females (Avenant and Nel 1998; Leighton et al. 2025), we subsequently built models for both the dry and wet seasons that included all habitat covariates and their interaction with sex (Table 2). We used mixed‐effects logistic regression (Harrison et al. 2018) to parametrize these models and estimate season‐specific coefficients and *p*‐values. Analyzes were performed in the R computing environment (version 3.6.5; R Core Team 2019) and JMP Pro (version 18, SAS Inc.). To verify that the more reduced seasonal datasets were not biased, we repeated the same modeling procedure with the full dataset of caracal locations.

**TABLE 2:** The presence of caracals in the Arava Valley as a function of sex and eight geophysical predictors.

Term	Wet season						Dry season					
	Estimate	Lower 95% CI	Upper 95% CI	*p*	Total effect	VIF	Estimate	Lower 95% CI	Upper 95% CI	*p*	Total effect	VIF
Sex[F]	−1.493e‐1	−1.040e+0	7.418e‐1	0.696	0.28	1.5	−9.024e‐2	−1.153e+0	9.729e‐1	0.842	0.65	2.0
Road distance	−1.597e‐5	−1.025e‐4	7.054e‐5	0.717	< 0.01	1.2	2.057e‐4	1.392e‐4	2.723e‐4	**< 0.001**	0.07	1.2
Trail distance	1.106e‐3	9.197e‐4	1.292e‐3	**< 0.001**	0.08	1.5	−4.897e‐4	−6.216e‐4	−3.579e‐4	**< 0.001**	0.03	1.7
Elevation	−2.736e‐2	−3.212e‐2	−2.260e‐2	**< 0.001**	0.66	1.0	−1.125e‐2	−1.399e‐2	−8.506e‐3	**< 0.001**	0.41	1.0
Slope	2.550e‐2	−6.025e‐3	5.702e‐2	0.113	0.02	1.0	−1.925e‐2	−4.271e‐2	4.206e‐3	0.108	0.04	1.0
Aspect	−1.285e‐3	−2.036e‐3	−5.330e‐4	**0.001**	0.01	1.1	−1.012e‐3	−1.589e‐3	−4.348e‐4	**0.001**	0.01	1.2
TPI	−1.523e‐1	−2.381e‐1	−6.653e‐2	**0.001**	0.24	1.8	5.088e‐2	1.060e‐2	9.116e‐2	**0.013**	0.40	2.2
Field distance	−1.373e‐5	−1.050e‐4	7.758e‐5	0.768	0.03	1.3	−2.813e‐4	−3.403e‐4	−2.224e‐4	**< 0.001**	0.24	1.2
Creek distance	−6.979e‐4	−8.826e‐4	−5.131e‐4	**< 0.001**	0.07	1.5	−2.445e‐4	−3.557e‐4	−1.333e‐4	**< 0.001**	0.02	2.0
Sex[F]*Road distance	−3.518e‐5	−1.219e‐4	5.150e‐5	0.426			9.253e‐5	2.612e‐5	1.589e‐4	**0.006**		
Sex[F]*Trail distance	6.178e‐5	−1.243e‐4	2.478e‐4	0.515			−4.332e‐5	−1.753e‐4	8.861e‐5	0.520		
Sex[F]*Elevation	−1.122e‐2	−1.597e‐2	−6.477e‐3	**< 0.001**			1.573e‐2	1.297e‐2	1.849e‐2	**< 0.001**		
Sex[F]*Slope	3.745e‐2	5.868e‐3	6.903e‐2	**0.020**			5.172e‐2	2.828e‐2	7.515e‐2	**< 0.001**		
Sex[F]*Aspect	−2.214e‐3	−2.966e‐3	−1.461e‐3	**< 0.001**			−1.418e‐3	−1.995e‐3	−8.401e‐4	**< 0.001**		
Sex[F]*Topography	−1.667e‐2	−1.024e‐1	6.910e‐2	0.703			−1.522e‐1	−1.925e‐1	−1.119e‐1	**< 0.001**		
Sex[F]*Field distance	1.796e‐4	8.807e‐5	2.712e‐4	**< 0.001**			−2.474e‐4	−3.059e‐4	−1.890e‐4	**< 0.001**		
Sex[F]*Creek distance	6.222e‐4	4.375e‐4	8.068e‐4	**< 0.001**			−2.035e‐4	−3.148e‐4	−9.221e‐5	**< 0.001**		

*Note:* Binomial mixed model estimate (±95% CI), *p*‐value, total effect, and VIF for each of the predictors and its interaction with sex during the wet (Oct‐Mar) and dry (Apr‐Sep) seasons. Effect size is evaluated using the total effect. Significant effects are in bold.

### 2.5 Diet Analysis

To determine the identity and frequency of items in caracal diets, we opportunistically collected fecal samples from areas of caracal captures and areas where collared individuals were frequently detected (Weisbein and Mendelssohn 1990). The samples did not necessarily reflect the dietary preferences of captured individuals. All samples were stored in bags and analyzed in a laboratory under a dissecting microscope, using standard methodology (Lindsay and Macdonald 1986). Undigested food items (i.e., bones, hair, feathers, scales, wings and plant material) were classified according to a species key specific to southern Israel as Dorcas gazelle ( *Gazella dorcas* ), rock hyrax ( *Procavia capensis* ), Cape hare, small rodent, domestic mammal, sand partridge ( *Ammoperdix heyi* ), passerine bird, domestic bird, reptile, insect and vegetation. We calculated the occurrence proportion of each food category relative to the entire sample, and present these results in percentages.

### 2.6 Recent Caracal Distribution

To assess the distribution patterns of caracals across southern Israel over the last four decades, we compiled and analyzed observational records collected by field rangers of the Israel Nature and Parks Authority (INPA) between 1979 and 2025. Between 1979 and 2008, animal sightings were manually registered and assembled in the INPA's database. Since 2008, rangers entered animal observation using a “Cyber Ranger” smartphone application including a spatial reference associated with each record. Observations were georeferenced and included incidental sightings during patrols, road surveys, and dedicated monitoring efforts. To quantify habitat associations, we calculated the Euclidean distance from each caracal observation location to the nearest creek or riverbed, based on the FFRivers spatial dataset (Grill et al. 2019).

### 2.7 Cape Hare Distribution

To understand whether caracal space use was driven by its main prey item, the Cape hare ( *Lepus capensis* ), we used a dataset of Cape hare observations georeferenced by INPA rangers, collected between 2010 and 2024. We used the Near function of ArcGIS to calculate the distance between each hare detection and the nearest river or creek according to the spatial FFRivers dataset (Grill et al. 2019). Within a polygon bounding all INPA hare detections, we subsequently generated an equal number of random points. We compared the mean and distribution of distances between observed and available locations to determine whether Cape hares were associated with creek and river features. We used a generalized regression model with gamma distribution to examine whether observed distances significantly differed from random. To examine possible seasonal distribution patterns, we subsequently used a *t*‐test (McDonald 2009) comparing mean distances between observed Cape hare locations in the wet and dry seasons.

## 3 Results

We captured 10 caracals, five males and five females, for which we obtained 3610 locations from 75 tracking days comprised of 24‐h monitoring sessions (Table S1). All tracked and observed individuals were adults. Mean area of observed use was larger for males (mean ± SE = 113.9 ± 25.5 km^2^) compared to females (mean ± SE = 46.1 ± 15.5 km^2^). This pattern remained significant after removing individuals with less than 100 locations (male mean = 128.7 km^2^, female mean = 68.6 km^2^).

### 3.1 Resource Selection Functions

Distances from fields and settlements were highly correlated. To eliminate collinearity in the predictors, we decided to retain distance from fields in the models because they were the more prevalent features in the landscape (all VIF values < 3; Table 2). The outputs of models describing caracal habitat selection revealed significant sex‐specific patterns.

**FIGURE 3:**
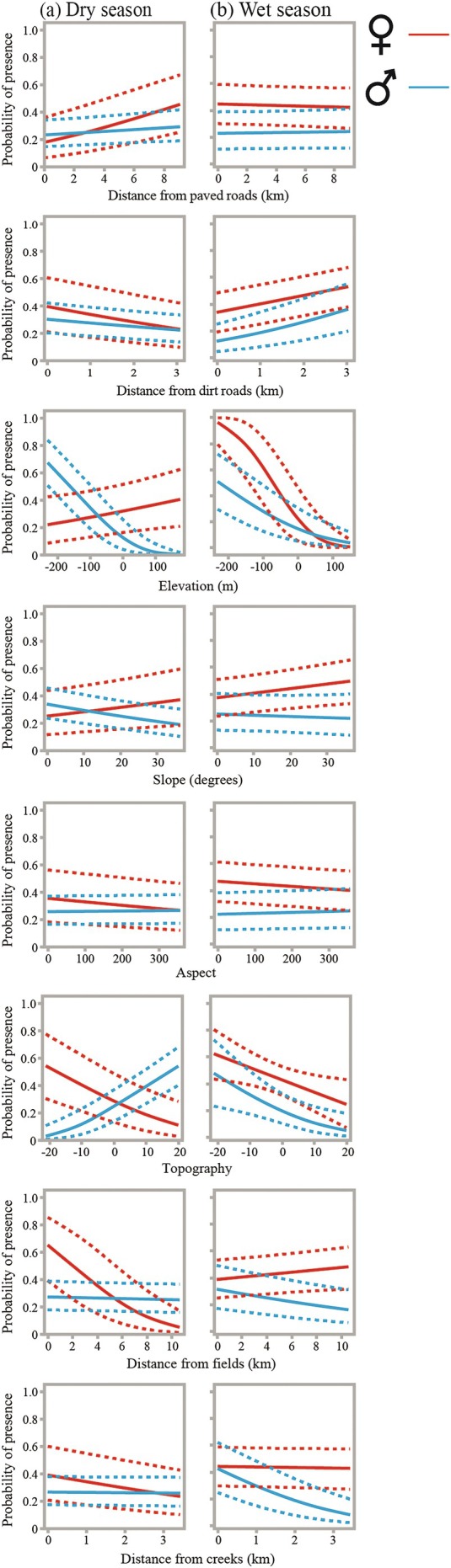
Predictions of generalized linear mixed models examining the spatial covariates predicting caracal space use in the Arava area. Graphs showing predicted probability of presence (±95% CI) for males (blue) and females (red) in the dry (a) and wet (b) seasons.

During the wet season, caracal space use was primarily driven by topography, elevation, and proximity to hydrological and linear features (Table 2, Figure 3). Probability of presence declined strongly with increasing elevation (*p* < 0.001) and TPI (*p* = 0.001), indicating preferential use of lower‐lying areas and topographic depressions. Caracals also selected locations closer to creeks (*p* < 0.001), consistent with increased use of these relatively productive habitats during the wet season. Distance to dirt trails was positively associated with use (*p* < 0.001), suggesting avoidance of trails, whereas distance to paved roads (*p* = 0.717) and fields (*p* = 0.768) had no detectable effects. Aspect showed a small but significant negative effect (*p* = 0.001), while slope was not significant (*p* = 0.113). Several sex‐specific responses were evident: females showed stronger avoidance of higher elevations (sex × elevation, *p* < 0.001), specific aspects (sex × aspect, *p* < 0.001), and greater distances from creeks (sex × creek distance, *p* < 0.001), as well as significant interactions with slope (*p* = 0.020) and distance from fields (*p* < 0.001), indicating finer‐scale habitat partitioning by females.

In the dry season, habitat selection shifted toward stronger responses to anthropogenic features and broader landscape structure. Caracals used areas farther from paved roads (*p* < 0.001) and closer to dirt trails (*p* < 0.001), indicating road avoidance coupled with increased use of minor tracks. Selection probability declined with increasing elevation (*p* < 0.001) and distance from creeks (*p* < 0.001) and fields (*p* < 0.001), suggesting concentration in low‐elevation areas near water sources and agricultural edges. In contrast to the wet season, TPI had a positive effect (*p* = 0.013), indicating increased use of relatively elevated or convex landforms under dry conditions. Aspect again had a consistent negative effect (*p* = 0.001), while slope remained non‐significant (*p* = 0.108). Sex‐specific interactions were pronounced, with females showing stronger responses to elevation, slope, topography, and distances from roads, fields, and creeks (all Sex × variable interactions *p* ≤ 0.006), highlighting increased sexual segregation in habitat use during the dry season (Table 2, Figure 3).

Results from the model including both the dry and wet season datasets were similar, with significant coefficients for interactions between sex and distance from roads (*p* < 0.001), sex and elevation (*p* < 0.001), sex and slope (*p* < 0.001), sex and aspect (*p* < 0.001), and sex and distance from creeks (*p* < 0.001; Table S2). We felt that the seasonal datasets provided valuable insights and thus decided to present them in the main text. The full dataset results are presented in Table S2.

### 3.2 Diet Analysis

**FIGURE 4:**
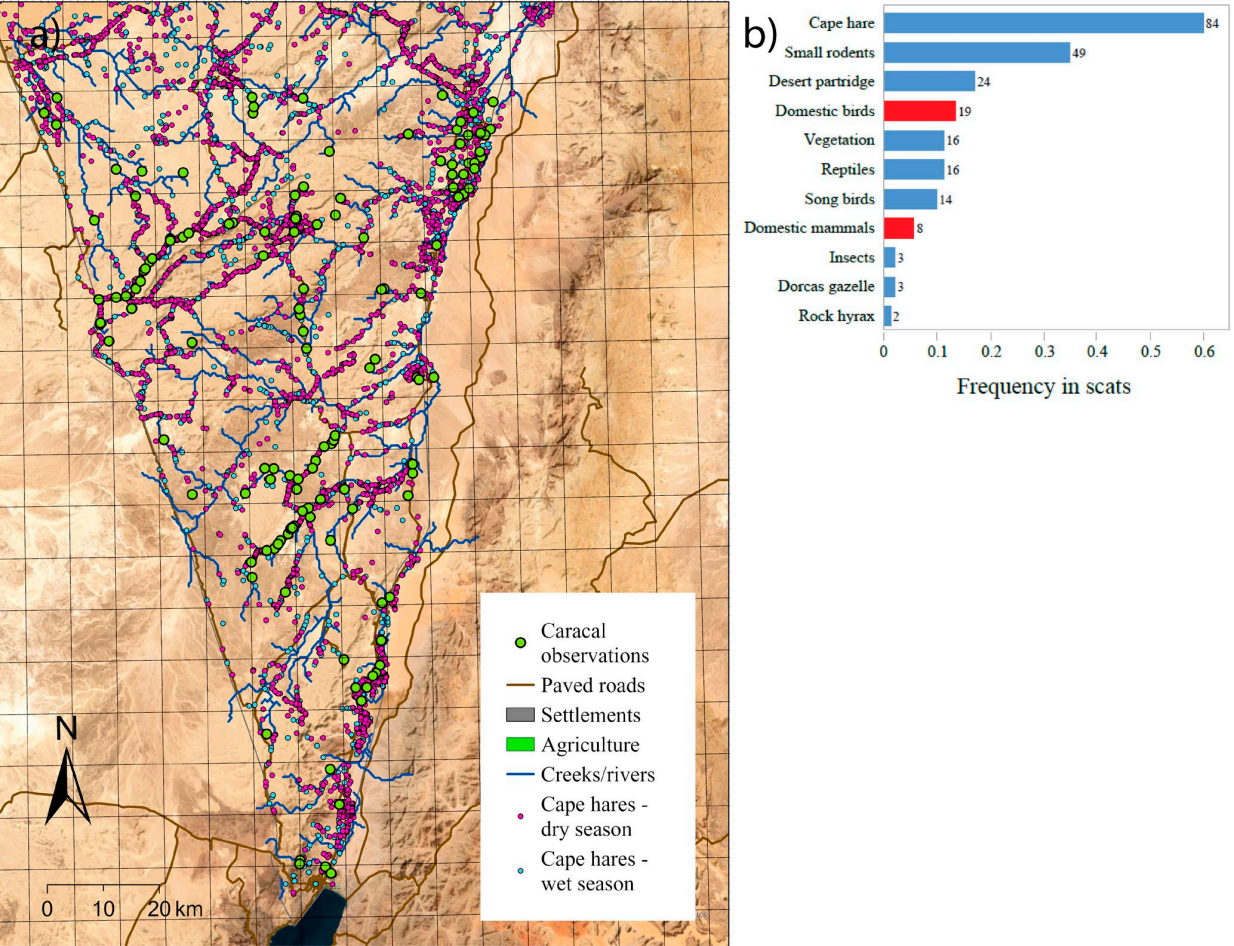
(a) Map of 159 caracal and 8144 cape hare observations collected by INPA rangers between 1979 and 2025 in southern Israel. (b) Occurrence frequency of 11 prey categories in 140 caracal scats collected during 1986–87 in the study area. Red bars denoted food items associated with human habitation. Further detail is available in Table S3.

We collected 140 scat samples between 1986 and 1987. The most frequently found item was the Cape hare (84 detections, 35.3% of scat samples) followed by small rodents (20.6%) and desert partridge (10.1%; Figure 4b). A total of 27 items (11.3%) were of anthropogenic nature, including the remains of domestic cats, goats, and donkeys (Table S3). These results should be interpreted with caution given the limitations of carnivore diet analysis based on scats (Chakrabarti et al. 2016).

### 3.3 Recent Caracal Distribution

To corroborate the RSF findings concerning creek preference, we compiled 159 caracal observations in the southern part of Israel made between 1979 and 2025. Some observations were made along roads, which may reflect the travel patterns of rangers who collected these data. The caracals were observed mostly along creek beds (mean distance to creek beds = 0.778 km; Figure 4).

### 3.4 Cape Hare Distribution

We compiled 8144 cape hare observations between 2010 and 2024 (Figure 4a) and compared them with 8000 randomly generated locations spanning the southern part of Israel. The mean (±SE) distance of observed hare locations from the nearest creek was 0.941 (±0.01) km, which is significantly shorter (Wald *χ* ^2^_1_ = 1250.6, *p* < 0.0001) than the mean random location distance of 1.477 (±0.015) km. This significant difference in distances from creeks between the observed and random locations suggests a strong preference of Cape hares for the proximity of creeks. Cape hare distances from creeks in the wet season (mean = 0.99 ± 0.01 km) were slightly higher compared to the dry season (mean = 0.90 ± 0.01 km; *t*‐test *p* < 0.01; Figure 4a).

## 4 Discussion

Our analysis of fine‐scale space use by tagged caracals near agricultural settlements in an arid area of southern Israel indicated that caracal movement is influenced by both topographic features and human infrastructure. These patterns were both season and sex specific. Dietary analysis revealed that this caracal population feeds mostly on cape hares and small rodents, with anthropogenic food items comprising a small proportion of its diet. Long‐term observational data further demonstrated that both caracals and their primary prey (Cape hares) were spatially associated with creek beds, suggesting that natural prey availability strongly drives caracal habitat selection. These findings highlight the ecological plasticity of a carnivore faced with a resource‐limited arid environment, while raising important considerations for its conservation in human‐modified landscapes.

Mean short‐term areas of use for male caracals in the Arava were approximately twice those of females, confirming previous findings of larger male home ranges in this species (Avenant and Nel 1998; Leighton et al. 2025). However, the absolute areas of use estimated here (Male mean = 113.9 km^2^; Female mean = 46.1 km^2^) are considerably smaller than home ranges reported for caracals in other arid regions. For example, caracal home ranges have been estimated at over 300 km^2^ in the Kalahari Desert (Bothma and Le Riche 1994) and over 1000 km^2^ in the steppe desert of Saudi Arabia (van Heezik and Seddon 1998). These differences likely reflect our limited tracking durations, as extended monitoring has been shown to substantially increase range estimates (van Heezik and Seddon 1998). As tracking periods lengthen, caracal ranges in the Arava may encompass a broader mosaic of habitat types, potentially expanding niche breadth and diet diversity (Leighton et al. 2025).

Patterns of habitat selection derived from RSF analyzes suggest that sex‐specific space use is structured less by overall range size and more by differential responses to topography, hydrology, and anthropogenic features. While both sexes favored lower elevations and areas closer to creeks, females showed significantly stronger associations with these features, particularly during the dry season. Sex‐specific interactions with elevation, topographic position, distance to fields, and proximity to creeks may indicate sexual segregation in habitat use, especially under drier conditions when resources are more spatially constrained. Contrary to expectations, slope did not exhibit a strong main effect for either sex; instead, its influence emerged through sex‐dependent responses, suggesting divergent use of terrain. These patterns are consistent with sex‐specific foraging and risk‐management strategies, whereby females may prioritize predictable resources and safer habitats—potentially linked to reproductive demands—while males range more broadly across heterogeneous landscapes, possibly reflecting greater tolerance of energetic or disturbance‐related costs.

Both male and female caracals demonstrated strong and consistent selection for proximity to creeks, with this association evident in both seasons and intensified during the wet season. In contrast, selection for proximity to agricultural fields was season‐ and sex‐dependent, with little evidence of field‐related selection during the wet season but a pronounced attraction during the dry season, particularly among females. These patterns reflect the highly dynamic nature of the southern Negev landscape, where localized precipitation and runoff can drive strong spatio‐temporal variation in vegetation and resource availability, especially during the wet season. The caracal's seasonal modulation in selection for the proximity of creeks and fields may be a response to this variability, providing further support for the ecological flexibility and adaptability to a wide range of conditions and levels of human disturbance (Leighton et al. 2020, 2024).

Selection for proximity to creeks and flatter creek beds likely reflects the higher likelihood of encountering Cape hares, the most frequent prey item in the Negev caracal's diets, as well as small rodents. This is supported by ranger observation data between 2010 and 2024, indicating a higher incidence of Cape hares in these areas. In addition to prey availability, these flat, lower areas are also characterized by lower elevations and more buffered climate conditions, potentially offering thermal relief from the desert heat during hot daytime periods. Together, these features may make such habitats especially valuable during periods of reduced resource availability, helping to explain their strong and persistent selection across seasons.

Our findings are in line with previous research depicting caracals as ecological generalists that maintain a reliance on natural prey while employing spatial strategies to navigate human‐modified landscapes. RSF analyzes indicate strong and consistent selection for natural landscape features such as creek beds across seasons, supporting their continued reliance on relatively undisturbed habitats. In contrast, responses to anthropogenic linear features were highly context dependent. Caracals showed clear avoidance of paved roads during the dry season, with weaker or absent effects during the wet season, and evidence of sex‐specific responses. Similarly, selection relative to dirt roads shifted seasonally, with avoidance during the wet season and increased use during the dry season. These patterns suggest that caracals discriminate between levels of human disturbance, avoiding major infrastructure while conditionally exploiting low‐intensity linear features when they may facilitate movement or access to resources. Such context‐dependent responses highlight the species' behavioral plasticity and capacity to persist across landscapes shaped by varying degrees of human activity (Ramesh et al. 2017; Serieys et al. 2023).

In line with dietary patterns documented in previous studies (Parchizadeh et al. 2023), the most frequent items in caracal diets were small mammals, especially Cape hares. The number of fecal samples did not allow us to examine seasonality in caracal diet. Observations of caracals feeding on prey such as domestic cats and captive animals from local enclosures were mostly concentrated in winter months (November to February; Weisbein and Mendelssohn 1990). A possible explanation for this is that during the colder season caracals focus their efforts on prey that are easier to capture and thereby minimize energy expenditure, occasionally assuming the risks presented by human infrastructure. Despite the caracal's proximity to human settlements, our dietary analysis revealed that anthropogenic food items constituted only 11.3% of their diet, highlighting the species' continued reliance on natural prey even in human‐modified landscapes. This may be a result of not accounting for digestibility in our diet analysis. Alternatively, this low proportion of human‐associated foods may suggest that caracals are maintaining their ecological function as native predators rather than shifting toward human subsidies, differing thereby from the more opportunistic carnivores like the Arabian wolf, golden jackal and red fox which often exploit anthropogenic resources (Assa 1990; Barocas et al. 2018; Lewin et al. 2021). The strong spatial association near creek beds between caracals and their primary prey, Cape hares, as documented by observations during the last four decades, further supports the conclusion that it is natural prey availability—not human food sources—that primarily drives caracal habitat selection in this arid ecosystem.

While our analysis provides valuable insight into the ecology of caracals in this hyperarid system, we acknowledge its limitations, including sample size and short tracking periods, which can pose limits to inference to short‐term space use rather than annual home ranges. Furthermore, we recognize the temporal disparity between the telemetry and diet data (1986–1987) and the long‐term ranger observational data (1979–2025). The latter dataset is also subject to spatial bias toward easily accessible areas, such as roads, reflecting the travel patterns of the rangers. Despite these disparities and biases, distinct datasets point toward a consistent ecological pattern. The RSF approach, which accounted for spatial covariates and individual variability, indicated strong and statistically significant selection for proximity to creek beds across both seasons. This finding aligns with the independent dietary analysis, which revealed a primary reliance on wild prey, particularly the Cape hare, with anthropogenic food items constituting only a small fraction of the diet (11.3%). Finally, while cautiously interpreting the spatially biased ranger data, the persistent spatial association recorded between caracals and creek beds (mean distance = 0.778 km) over four decades provides qualitative support for our conclusion that natural prey availability, rather than human subsidies, drives caracal habitat selection in this resource‐limited environment.

Southern Israel has a protected area coverage considered sufficient to support local biodiversity and maintain viable native carnivore populations (Rotem and Weil 2014). A promising future research direction would be to reassess this assumption by examining more recent patterns of habitat selection and space use in caracals and their interactions with native and commensal carnivore species, under ongoing landscape change driven by the expansion of agricultural activity. Such studies could benefit from current technological advances in movement ecology (Kays et al. 2015) and remote sensing, including detailed contemporary land use and habitat spatial layers. Understanding how caracals navigate the ecological interface between protected and human‐modified areas can inform more targeted conservation planning. Ultimately, these insights are essential for fostering coexistence between local native wildlife and the expanding agricultural development in arid regions.

## Author Contributions

**Adi Barocas:** formal analysis (equal), methodology (equal), writing – original draft (lead). **Yaron Weisbein:** conceptualization (equal), data curation (equal), investigation (equal). **Eli Geffen:** formal analysis (equal), methodology (equal), writing – original draft (equal), writing – review and editing (equal).

## Ethics Statement

All field procedures for this study were in accordance with the Israeli Nature and Parks Authority (NPA) guidelines and regulations. Annual permit numbers: 063126/1986 and 063126/1987. Animal welfare concerns were taken into account during trapping and tagging sessions.

## Conflicts of Interest

The authors declare no conflicts of interest.

## Supporting information


**Data S1:** ece373117‐sup‐0001‐supinfo.docx.

## Data Availability

The data files attached with the submission contain caracal locations, datasets for the resource selection function analyzes, and Cape hare observation locations.
