# Supplementary material for: Creek Beds and Cape Hares: Spatial and Seasonal Determinants of Caracal Movement and Diet in a Hyperarid Desert Ecosystem
Source: Ecol Evol. 2026 Feb 17;16(2):e73117. doi: 10.1002/ece3.73117 (PMC12912937; doi:10.1002/ece3.73117)

**Creek beds and cape hares:** **spatial and seasonal determinants of caracal movement and diet in a hyperarid desert ecosystem**

Adi Barocas^1,2^*, Yaron Weisbein^3^*, Eli Geffen^4^

*1. Hula Research Center, Department of Animal Sciences, Tel-Hai College, Israel*

*2. MIGAL–Galilee Research Institute, Kiryat Shmona, Israel.3 - Israel Nature and Parks Authority, Southern District, Beer Sheva, 84215, Israel*

*3. Ze'ev Brande 5, Petah Tikva 4960041, Israel*

*4. School of Zoology, Tel Aviv University, Tel Aviv 69978, Israel*

*** equal contribution

**Tables**

**Table S1.** Summary data of the 10 caracal individuals fitted with VHF collars and monthly tracked during 1986 and 1987.

| ID | Sex | Year | Dry | Wet | Days tracked | Successful fixes | 90% KUD (km^2^) | 95% KUD (km^2^) | 90% MCP (km^2^) | 95% MCP (km^2^) |
| --- | --- | --- | --- | --- | --- | --- | --- | --- | --- | --- |
| Alberto | Male | 1986-87 | + | + | 9 | 431 | 101.1 | 121.7 | 70.2 | 77.1 |
| Anony | Male | 1986-87 | + | + | 7 | 339 | 67.0 | 96.1 | 40.1 | 61.6 |
| Arava | Female | 1987 | + | + | 2 | 99 | 33.6 | 44.2 | 8.5 | 9.6 |
| Beber | Male | 1986-87 | + | + | 14 | 679 | 180.5 | 228.8 | 171.1 | 178.0 |
| Fritz | Male | 1986-87 |  | + | 2 | 95 | 55.1 | 80.0 | 20.8 | 22.8 |
| Michal | Female | 1986 | + | + | 7 | 339 | 50.5 | 66.3 | 19.2 | 19.2 |
| Oog | Male | 1986-87 | + | + | 7 | 344 | 165.9 | 209.3 | 144.0 | 144.0 |
| Sany | Female | 1987 | + | + | 3 | 146 | 27.4 | 41.0 | 8.3 | 8.3 |
| Sheiz | Female | 1986 |  | + | 1 | 49 | 15.3 | 21.6 | 0.5 | 0.7 |
| Zilpa | Female | 1986-87 | + | + | 23 | 1089 | 103.9 | 132.4 | 82.5 | 102.9 |

**Table S2.** The presence of caracals in the Arava Valley as a function of sex and eight geophysical predictors. Binomial mixed model estimate (± 95% CI), p-value, and total effect for each of the predictors and its interaction with sex. This model combined the data for both dry and wet seasons. Caracal identity and date were set as random factors. Effect size was evaluated using the total effect. Significant effects are in bold.

| Term | Estimate | Lower 95% CI | Upper 95% CI | P | Total effect |
| --- | --- | --- | --- | --- | --- |
| Sex[F] | -1.553e+0 | -2.370e+0 | -7.364e-1 | **0.001** | 0.49 |
| Road distance | -8.701e-2 | -8.398e-1 | 6.658e-1 | 0.797 | 0.03 |
| Trail distance | 1.287e-4 | 7.859e-5 | 1.787e-4 | **<0.001** | 0.01 |
| Elevation | 1.154e-4 | 1.760e-5 | 2.132e-4 | **0.021** | 0.51 |
| Slope | -1.416e-2 | -1.637e-2 | -1.195e-2 | **<0.001** | 0.03 |
| Aspect | 2.633e-4 | -1.763e-2 | 1.816e-2 | 0.977 | 0.01 |
| TPI | -1.121e-3 | -1.561e-3 | -6.808e-4 | **<0.001** | 0.38 |
| Field distance | -3.389e-3 | -3.885e-2 | 3.207e-2 | 0.851 | 0.16 |
| Creek distance | -2.354e-4 | -2.814e-4 | -1.894e-4 | **<0.001** | 0.05 |
| Sex[F]*Road distance | -4.644e-4 | -5.557e-4 | -3.731e-4 | **<0.001** |  |
| Sex[F]*Trail distance | 4.986e-5 | -2.293e-7 | 9.996e-5 | 0.051 |  |
| Sex[F]*Elevation | -1.769e-4 | -2.746e-4 | -7.924e-5 | **<0.001** |  |
| Sex[F]*Slope | 6.577e-3 | 4.361e-3 | 8.793e-3 | **<0.001** |  |
| Sex[F]*Aspect | 3.631e-2 | 1.843e-2 | 5.419e-2 | **<0.001** |  |
| Sex[F]*Topography | -1.810e-3 | -2.250e-3 | -1.369e-3 | **<0.001** |  |
| Sex[F]*Field distance | -1.107e-1 | -1.461e-1 | -7.527e-2 | **<0.001** |  |
| Sex[F]*Creek distance | -9.845e-5 | -1.442e-4 | -5.266e-5 | **<0.001** |  |

**Table S3.** Identities and relative frequencies of identifiable prey items found in caracal feces of the Northern Arava in Israel.

| Species | Scientific name | Taxon | Number of fecal samples | Relative frequency |
| --- | --- | --- | --- | --- |
| Cape hare | Lepus capensis | Mammals | 84 | 35.29 |
| Gerbilidae |  | Mammals | 14 | 5.88 |
| Common spiny mouse | *Acomys cahirinus* | Mammals | 2 | 0.84 |
| Other rodents |  | Mammals | 33 | 13.87 |
| Rock hyrax | Procavia capensis | Mammals | 2 | 0.84 |
| Dorcas gazelle | *Gazella dorcas* | Mammals | 3 | 1.26 |
| Domestic cat | *Felis silvestris catus* | Mammals | 6 | 2.52 |
| Goat | *Capra aegagrus hircus* | Mammals | 1 | 0.42 |
| Donkey | *Equus africanus asinus* | Mammals | 1 | 0.42 |
| Sand partridge | *Ammoperdix heyi* | Birds | 24 | 10.08 |
| Passerine birds |  | Birds | 14 | 5.88 |
| Domestic turkey | *Meleagris sp* | Birds | 3 | 1.26 |
| Domestic chicken | *Gallus gallus domesticus* | Birds | 10 | 4.2 |
| Rock pigeon | *Columba Livia* | Birds | 6 | 2.52 |
| Agamid lizard | *Uromastyx aegyptia* | Reptiles | 13 | 5.46 |
| Nidua fringe-fingered lizard | *Acanthodactylus scutellatus* | Reptiles | 2 | 0.84 |
| Unidentified snake |  | Reptiles | 1 | 0.42 |
| Unidentified insects |  | Insects | 3 | 1.26 |
| Unidentified plants |  | Plants | 16 | 6.72 |

**Figures**

**Figure S1.** Home range estimates, random and observed locations of two individual caracals followed for nine and 14 days in Israel’s Negev desert during 1986 and 1987.


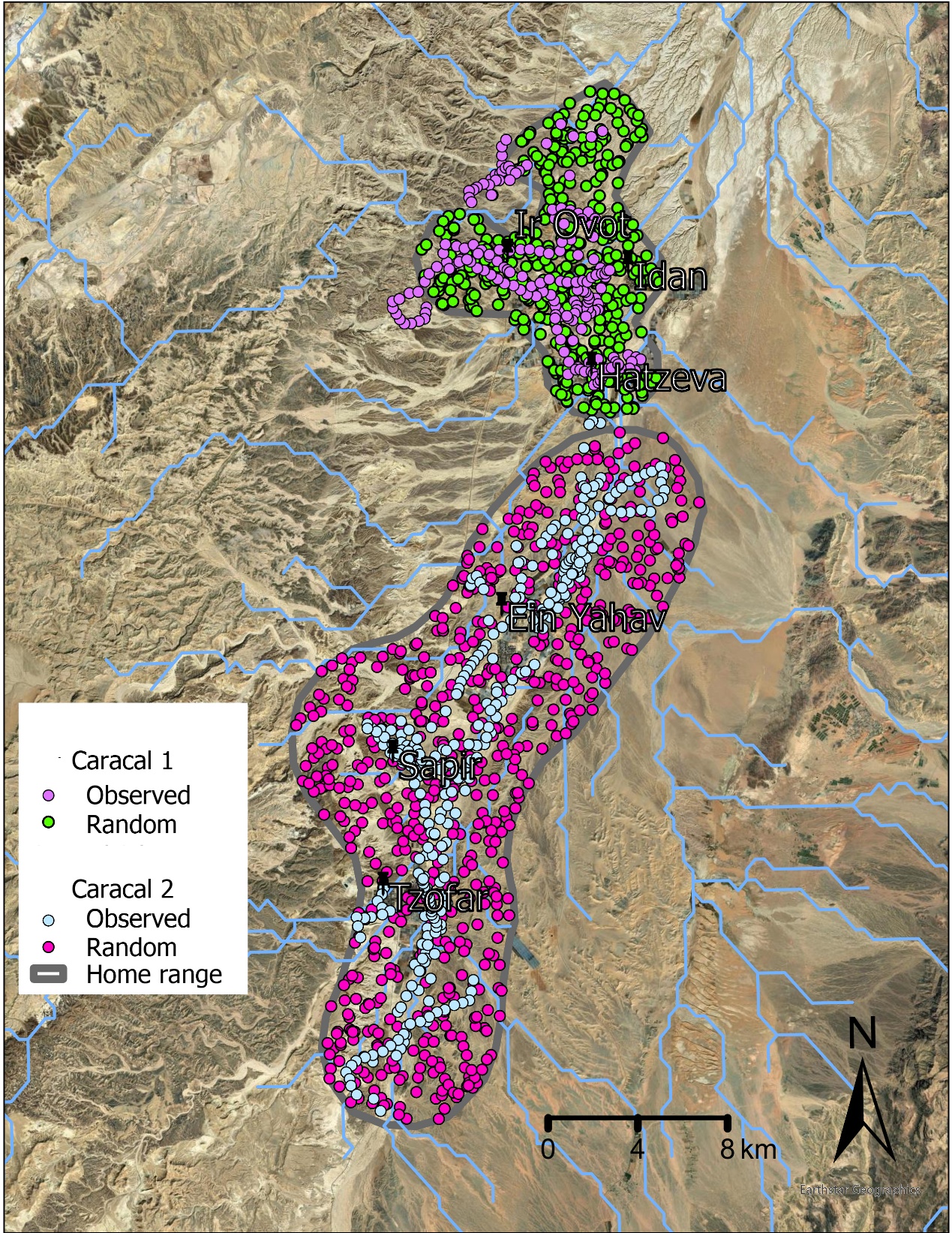

Supplement: Supplementary file 1 — Data S1: ece373117‐sup‐0001‐supinfo.docx. [file ECE3-16-e73117-s001.docx]
